# Intravenous Delivery of *piggy*Bac Transposons as a Useful Tool for Liver-Specific Gene-Switching

**DOI:** 10.3390/ijms19113452

**Published:** 2018-11-02

**Authors:** Shingo Nakamura, Masayuki Ishihara, Satoshi Watanabe, Naoko Ando, Masato Ohtsuka, Masahiro Sato

**Affiliations:** 1 Division of Biomedical Engineering, National Defense Medical College Research Institute, Saitama 359-8513, Japan; ishihara@ndmc.ac.jp (M.I.); naoandokoro@gmail.com (N.A.); 2 Animal Genome Unit, Institute of Livestock and Grassland Science, National Agriculture and Food Research Organization (NARO), 2 Ikenodai, Tsukuba, Ibaraki 305-0901, Japan; kettle@affrc.go.jp; 3 Division of Basic Medical Science and Molecular Medicine, School of Medicine, Tokai University, Kanagawa 259-1193, Japan; masato@is.icc.u-tokai.ac.jp; 4 Section of Gene Expression Regulation, Frontier Science Research Center, Kagoshima University, Kagoshima 890-8544, Japan; masasato@m.kufm.kagoshima-u.ac.jp

**Keywords:** Cre/*loxP*, diphtheria toxin-A chain, EGFP, hepatic disorder, hydrodynamics-based gene delivery, in vivo gene delivery, liver, *piggy*Bac transposon

## Abstract

Hydrodynamics-based gene delivery (HGD) is an efficient method for transfecting plasmid DNA into hepatocytes in vivo. However, the resulting gene expression is transient, and occurs in a non-tissue specific manner. The *piggy*Bac (PB) transposon system allows chromosomal integration of a transgene in vitro. This study aimed to achieve long-term in vivo expression of a transgene by performing hepatocyte-specific chromosomal integration of the transgene using PB and HGD. Using this approach, we generated a novel mouse model for a hepatic disorder. A distinct signal from the reporter plasmid DNA was discernible in the murine liver approximately two months after the administration of PB transposons carrying a reporter gene. Then, to induce the hepatic disorder, we first administered mice with a PB transposon carrying a CETD unit (loxP-flanked stop cassette, diphtheria toxin-A chain gene, and poly(A) sites), and then with a plasmid expressing the Cre recombinase under the control of a liver-specific promoter. We showed that this system can be used for in situ manipulation and analysis of hepatocyte function in vivo in non-transgenic (Tg) animals.

## 1. Introduction

The production of transgenic (Tg) animals has been one of the most powerful tools for exploring the function of genes of interest (GOIs) for analyzing pathogenic mechanisms and for developing therapeutic approaches [1]. However, this is time-consuming, labor-intensive, and expensive. To bypass this step, intravenous injection of naked DNA (usually plasmids) (or DNA usually complexed with delivery vehicles (e.g., cationic lipids)) or the direct administration of DNA using surgical techniques into tissues/organs has often been employed [2]. Gene delivery via a plasmid, however, is most commonly transient [3].

In animal experiment, tail vein injection of DNA or RNA is simple and convenient for gene delivery into a living organism. Liver-targeted hydrodynamics-based gene delivery (HGD) is an effective and safe method for gene delivery via non-viral vectors and has become one of the most commonly used techniques for assessing gene function in murine liver [4,5]. Hydrodynamics-based gene delivery uses the rapid injection of a relatively large volume of solution containing naked plasmid DNA and has been considered to be the most effective approach for delivering foreign genes into murine liver [2]. Unfortunately, gene expression in the liver is generally transient because the naked plasmid DNA introduced by HGD is often refractory to chromosomal integration into liver genome [2]. Furthermore, HGD causes transfection of other organs (such as lung and kidney) besides the liver, since it is mediated by tail vein injection [4]. If a researcher wants to express a GOI only in the liver using HGD, the use of a liver-specific promoter for driving GOI expression is a promising approach. However, most liver-specific promoters identified to date have weaker promoter activity than the widely used virus-derived promoters such as SV40 early or cytomegalovirus (CMV) promoter, which often makes it difficult to use liver-specific promoters to achieve stronger expression of a GOI.

There are several ways to enhance the activity of a tissue-specific but transcriptionally weak promoter [6,7,8,9,10,11]. For example, Nettelbeck et al provided a two-step amplification (TSTA) system, by which strong expression of GOI was induced by a strong promoter that had been activated through binding to the transcriptional activator produced from a weak promoter [12]. We also established a similar two-step approach for enhancing the expression of a GOI by employing the Cre/*loxP*-based gene switching system [13]. In detail, we first constructed a plasmid (tentatively called “A” here) carrying a strong promoter, *loxP*-flanked stop fragment, and a GOI. When this plasmid is introduced into a cell, *loxP*-flanked stop fragment (but not GOI) is strongly expressed under the strong promoter. However, when cells are co-transfected with plasmid A together with plasmid (tentatively called “B” here, which carries a Cre gene whose expression is controlled by a weak promoter), Cre protein derived from plasmid B should remove the *loxP*-flanked stop fragment in plasmid A, resulting in the initiation of GOI expression under the strong promoter present in plasmid A. Indeed, we observed enhanced expression of the GOI under these conditions. Furthermore, we achieved liver-specific but transient expression of the GOI in vivo by co-transfection of plasmid A and a plasmid conferring Cre expression under a liver-specific promoter [14].

The *piggy*Bac (PB) system derived from the cabbage looper moth *Trichoplusia ni* [15] is a one of the transposon–transposase system for efficient genetic modification of mammalian cells [16,17]. The PB transposase recognizes transposon-specific inverted terminal repeat sequences (ITRs) located on both ends of the transposon vector and efficiently integrates transgenes into the host genome [17]. However, the PB-mediated gene delivery system results in random integration of transgenes, leading to occasional transgene silencing, insertional mutagenesis, and positional variegation, probably as a result of transgene silencing [18,19]. These properties are especially important when this vector system is used for therapeutic gene transfer applications. The PB-based gene delivery has been reported to confer efficient chromosomal integration of GOIs in various types of in vitro cultured cell [20,21], generation of transgenic mice [20], gene discovery via insertional mutagenesis [22], and production of inducible pluripotent stem (iPS) cells [23,24,25,26]. Furthermore, Nakanishi et al demonstrated long-term gene expression of a GOI in vivo after HGD using PB-related vectors [27]. To our knowledge, however no reports of the achievement of long-term and tissue-specific expression of a GOI in vivo have been published. In this study, we examined whether HGD can be used in combination with a PB system to enable chromosomal integration of GOI for long-term expression and its tissue (hepatocytes)-specific gene-switching. We also tested whether this established technology can be used for the creation of mouse models for hepatic disorder caused by the conditional ablation of hepatocytes.

## 2. Results

### 2.1. HGD with PB Transposons Confers Continuous Expression of GOI in Murine Liver

**Figure 1:**
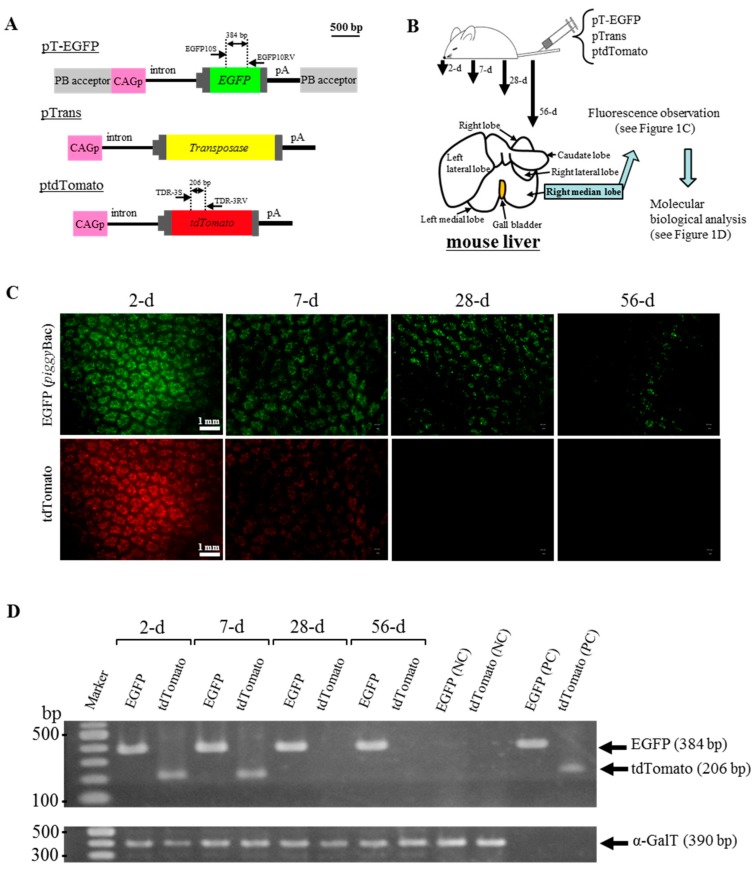
Hydrodynamics-based gene delivery and PB transposon system enable long-term gene expression in murine liver. (**A**) Structure of plasmid vectors used. The position of primers is shown above each plasmid. CAG, CMV enhancer + chicken β-actin promoter; intron, second intron of rabbit β-globin gene; EGFP, cDNA for enhanced green fluorescent protein; tdTomato, cDNA for tandem dimeric Tomato; pA, poly(A) sites; PB acceptor, acceptor site in PB system; (**B**) Schematic representation of experimental outline. Two PB-related plasmids (pT-EGFP and pTrans) and a non-PB plasmid ptdTomato are injected into adult ICR male mice via the tail vein by HGD. On 2, 7, 28, and 56 days after gene delivery, right median lobe of the liver is dissected for fluorescence observation and molecular biological analyses; (**C**) Fluorescent images of the right median lobe of the liver dissected 2, 7, 28, and 56 days (d) after gene delivery. tdTomato-derived fluorescence was observable on the specimens 2 to 7 days after gene delivery, whereas EGFP-derived fluorescence was still observed on the specimens 28 to 56 days after it; (**D**) PCR analysis of genomic DNA isolated from the right median lobe of a liver dissected 2, 7, 28, and 56 days (d) after gene delivery. The same amounts of genomic DNA were PCR-amplified using primers for detection of the endogenous α-GalT gene and used as internal controls. Negative control (NC), genomic DNA from intact mouse tail used as a negative control; Positive control (PC), plasmids pT-EGFP and ptdTomato (5 ng) used as positive controls for *EGFP* and *tdTomato* cDNA.

To examine whether PB transposons introduced into murine liver through HGD can guarantee the long-term expression of a GOI (*EGFP* cDNA in this case), adult Institute of Cancer Research (ICR) male mice were intravenously injected with a solution containing two PB-related vectors (pT-EGFP and pTrans; Figure 1A) and a non-PB vector ptdTomato (Figure 1A) using HGD. After HGD, the right median lobes in the liver of these treated mice were sampled 2, 7, 28, and 56 days after gene delivery, as shown in Figure 1B, and inspected for fluorescence under a fluorescence dissecting microscope. Enhanced green fluorescent protein (EGFP)-derived green fluorescence derived from PB vector pT-EGFP was still detected on the samples isolated 56 days after HGD (“EGFP” panels in Figure 1C and Figure S1). In contrast, tdTomato-derived red fluorescence derived from non-PB vector ptdTomato was undetectable on the samples isolated 28 days or more after HGD (“tdTomato” panels of Figure 1C). Similarly, polymerase chain reaction (PCR) analysis using genomic DNA isolated from the right median lobes demonstrated the presence of *EGFP* cDNA on the samples isolated from 2 to 56 days after HGD (Figure 1D). However, *tdTomato* cDNA was only detectable on the samples isolated 2 to 7 days after HGD (Figure 1D). These results indicate that PB-based gene delivery is more effective for long-term gene expression of a GOI in murine liver than transfection with non-PB plasmid DNA. 

### 2.2. Liver-Specific Gene Switching In Vivo Using PB and Cre/loxP Systems

**Figure 2:**
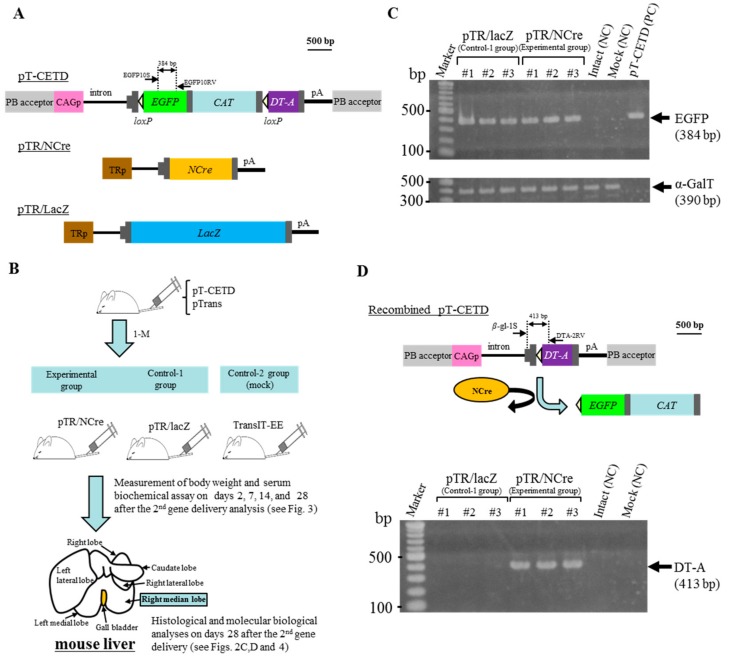
Hydrodynamics-based gene delivery and PB transposon system enable the creation of disease models for liver dysfunction. (**A**) Structure of plasmid vectors used for the creation of liver disease model mice. The position of primers is shown above each plasmid; (**B**) Schematic representation of experimental outline for the creation of mice with liver dysfunction. Adult ICR males are first subjected to HGD with a solution containing pT-CETD and pTrans. One month later, they are then subjected to the second HGD with a solution containing pTR/NCre (experimental group) or pTR/lacZ (control-1 group). Males are also mock-injected as control-2 group. After the second gene delivery and mock injection, blood is collected from these mice on the indicated days. Twenty-eight days after the second gene delivery and mock injection, mice are subjected to blood collection and then sacrificed to dissect their liver (right median lobe) for pathological and molecular biological analyses; (**C**) PCR analysis of genomic DNA isolated from the HGD-treated males in the experimental and control-1 groups. For each group, three females (#1 to #3) were analyzed. Genomic DNA (approximately 5 ng) was PCR-amplified using primers recognizing *EGFP* cDNA in pT-EGFP. Simultaneously, the same amounts of DNA were PCR-amplified using primers for detection of the endogenous α-GalT gene and used as internal controls. Intact (NC), genomic DNA from intact mouse liver used as a negative control; Mock (NC), genomic DNA isolated from mice in the control-2 group; PC, plasmid pT-CETD (5 ng) used as positive controls for *EGFP* cDNA sequence. Marker, 100-bp ladder markers; (**D**) Structure of pT-CETD after Cre-mediated recombination (upper panel) and PCR analysis of genomic DNA isolated from the females in the experimental and control-1 groups 28 days after the second gene delivery (lower panel). As shown in the upper panel, Cre protein provided from pTR/NCre acts to remove the *loxP*-flanked sequences in pT-CETD and finally allows the *DT-A* gene to be expressed by the upstream CAG. The *DT-A* gene after recombination can be detected by PCR using β-gl-1S and DTA-2RV primer set, as shown in the upper panel. Intact (NC), genomic DNA from intact mouse liver used as a negative control; Mock (NC), genomic DNA isolated from mice in the control-2 group. Marker, 100-bp ladder markers.

**Figure 3:**
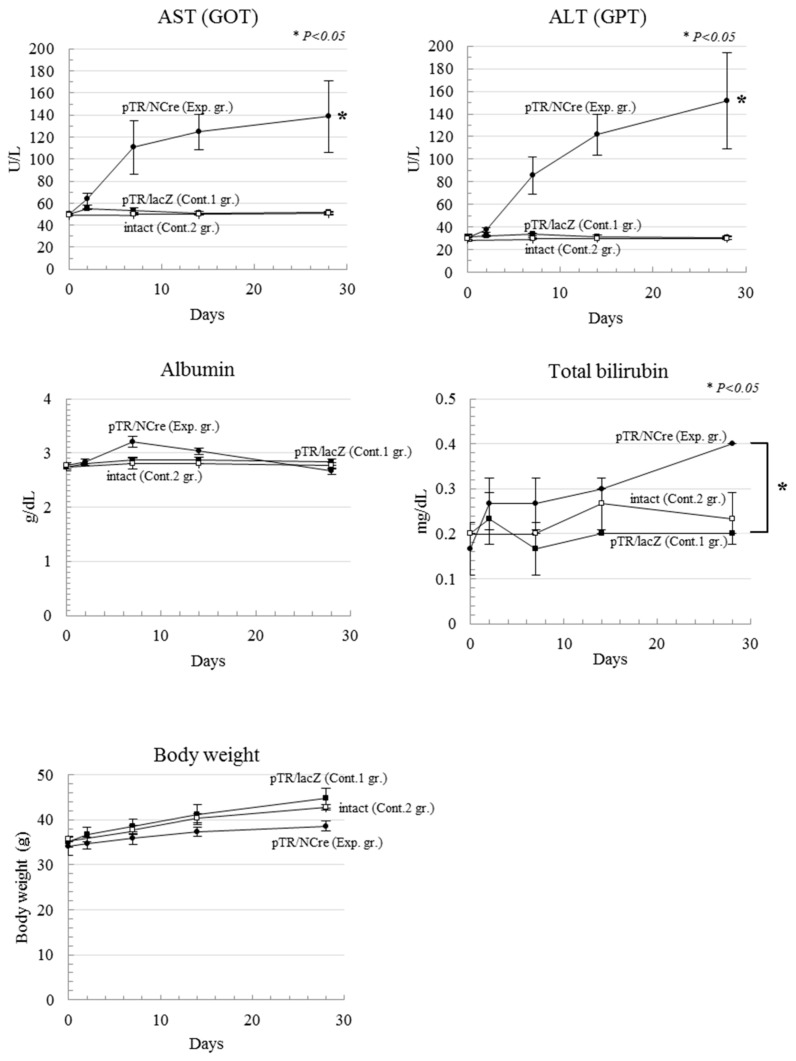
Hydrodynamics-based gene delivery and PB transposon system enable the creation of disease models for liver dysfunction: abnormal serum biochemical parameters and reduced body weight in the pT-CETD-incorporating males assayed 2, 7, 14, and 28 days after the second HGD with pTR/NCre (experimental group (Exp.gr.)) or pTR/lacZ (control-1 group (Cont.1 gr.)). Intact ICR males (in control-2 group (Cont. 2 gr.); with mice of the same age as in the experimental group) were also analyzed on 0, 7, 14, and 28 days. The level of significance set at *p* < 0.05 marked with an asterisk. Values are expressed as mean ± standard deviation among three mice tested for each group. AST, aspartate aminotransferase; ALT, alanine aminotransferase.

In previous experiments, we found that HGD coupled with the PB system allows long-term expression of a GOI in organs/tissues of a living organism, probably due to stable chromosomal integration of the GOI. This prompted us to test whether gene expression can be manipulated in the liver by administering a vector carrying a recombinase gene, whose expression is regulated under a liver-specific promoter. We first intravenously administered a solution containing pTrans (Figure 1A; a plasmid conferring PB transposase expression under systemic promoter CAG) and pT-CETD [Figure 2A; a PB-based plasmid carrying “CETD” comprising CAG, *loxP*-flanked *EGFP* cDNA, chloramphenicol acetyltransferase (*CAT*) gene, diphtheria toxin-A chain (*DT-A*) gene, and poly(A) sites; both ends of CETD are surrounded by PB acceptor sites] to six adult ICR males by HGD, as shown schematically in Figure 2B. One month after HGD, some hepatocytes were expected to possess CETD components in their chromosomes, as previously shown in the upper panel of Figure 1D. Three of these treated mice were again intravenously administered a TransIT-EE-based solution containing pTR/NCre (Figure 2A), a vector carrying the Cre gene whose expression is regulated by a liver-specific transthyretin promoter (TR), which were defined as an “experimental group” (Figure 2B). The other remaining three mice were also administered a TransIT-EE-based solution containing pTR/lacZ (Figure 2A), a vector carrying the lacZ (β-galactosidase) gene whose expression is regulated by TR, which were defined as “control-1 group” (Figure 2B). Moreover, adult ICR mice (*n* = 3) were mock-injected with TransIT-EE alone, being defined as “control-2 group” (Figure 2B). After the second gene delivery with pTR/NCre or pTR/lacZ, serum was collected on 2, 7, 14, and 28 days and, at the same time, body weight was measured (as shown in Figure 3). Serum was also collected from mice in the control-2 group. Twenty-eight days after the second gene delivery, the right median lobe of the liver was dissected for molecular biological and histological analyses (Figure 2B).

We first assessed the presence of *EGFP* cDNA included in the introduced pT-CETD by PCR using genomic DNA isolated from the right median lobe. For each group, three mice (#1 to #3) were subjected to the analyses. PCR analysis demonstrated the presence of *EGFP* cDNA in all of the samples tested (Figure 2C), suggesting chromosomal integration of pT-CETD in the liver of the tested animals. Next, we examined whether pTR/NCre introduced as the second gene delivery can excise the *loxP*-flanked *EGFP* cDNA + *CAT* gene from the chromosomally integrated CETD, as shown in the upper panel of Figure 2D. If expression of pTR/NCre occurs in hepatocytes carrying CETD components in their genome, NCre protein would recognize the *loxP* site in the CETD components and subsequently remove the *loxP*-flanked *EGFP* cDNA + *CAT* gene. This Cre-mediated excision leads to generation of a recombined pT-CETD, in which the *DT-A* gene has been placed immediately downstream of the CAG (upper panel of Figure 2D). PCR of genomic DNA using primers (β-gl-1S and DTA-2RV; upper panel of Figure 2D) demonstrated the presence of the recombined pT-CETD in all of the samples transfected with pTR/NCre (but not with pTR/lacZ) (lower panel of Figure 2D). Notably, we observed the presence of *EGFP* cDNA in the pTR/NCre-treated females (see Figure 2C). Thus, the Cre-mediated excision of the *loxP*-flanked *EGFP* cDNA + *CAT* gene shown in the pTR/NCre-treated mice may occur in a mosaic fashion: namely, some pT-CETD-incorporated hepatocytes exhibited Cre-mediated excision of the *loxP*-flanked sequences, but others did not. 

### 2.3. Serum Abnormality after DT-A Expression from Recombined pT-CETD

As shown in Figure 2B, serum was collected on 2, 7, 14, and 28 days and, at the same time, body weight was measured after the second gene delivery with pTR/NCre (as an experimental group) or pTR/lacZ (as control-1 group). Similarly, serum collection and measurement of body weight were performed for mice in the control-2 group. When serum biochemistry profiles were first assessed using blood samples, elevated levels of aspartate transaminase (AST) and alanine transaminase (ALT) in the experimental group were identified compared with those in control groups-1 and -2 (Figure 3). Notably, the level of AST was remarkably higher than that of ALT in the experimental group. The level of albumin tended to decrease with a peak at seven days after the second gene delivery with pTR/NCre (Figure 3). The level of total bilirubin increased from around seven days after the second gene delivery with pTR/NCre (Figure 3). Increase in body weight was also impaired in the experimental group: for example, means of body weight measured at 28 days after the second gene delivery with pTR/NCre were 86.0% and 90.2% of that in control groups-1 and -2, respectively (Figure 3).

### 2.4. Pathological Abnormality in Liver after DT-A Expression from Recombined pT-CETD

**Figure 4:**
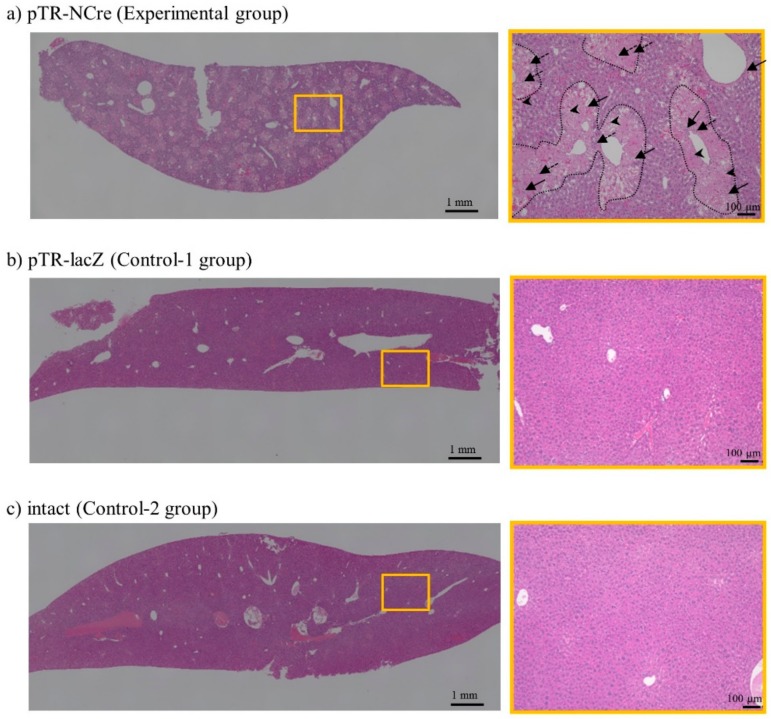
Hydrodynamics-based gene delivery and PB transposon system enable the creation of disease models for liver dysfunction. Pathological analysis of the pT-CETD-incorporated females assayed 28 days after the second HGD with pTR/NCre (experimental group) or pTR/lacZ (control-1 group). These data are representative of three animals. **Left panels**, low-magnification images of dissected liver from mice (#1) in the experimental (**a**), and control-1 (**b**), and -2 groups (**c**). Abnormality was remarkable throughout the entire lobe in the experimental group. In contrast, liver in the control-1 and -2 groups remained normal. **Right panels**, images highly magnified from the boxes in the experimental (**a**), and control-1 (**b**), and -2 groups (**c**) shown in the **left panels**. Note the mixture of necrotic and intact areas in the experimental group (**a**). In particular, focal necrosis (enclosed by dotted lines) and inflammation were remarkable. Representative lymph corpuscle, enlarged Kupffer cells, and eosinophil granulocyte are shown by arrows, dotted arrows and arrowheads, respectively. The necrotic portion was more eosinophilic and enlarged.

Twenty-eight days after the second gene delivery with pTR/NCre (as an experimental group) or pTR/lacZ (as control group-1), liver (right median lobe) was dissected and subjected to pathological analysis using hematoxylin and eosin (H&E)-stained specimens. Similarly, liver dissection was performed for mice in the control-2 group 28 days after the administration of TransIT-EE alone. In the experimental group, a whole lobe of liver exhibited pathological lesions (left panel of Figure 4a), which were associated with enlarged hepatocytes, focal necrosis, and inflammation (right panel of Figure 4a). In detail, the necrotic portion was more eosinophilic and enlarged (arrows within dotted lines in right panel of Figure 4a). These pathological features resemble hepatic lesions in hepatitis, and appear to reflect a severe condition potentially leading to cirrhosis. In contrast, the liver in controls-1 and -2 remained normal morphologically (Figure 4b,c). Thus, the lesions shown in the experimental group were indeed a result of conditional ablation of hepatocytes induced by a Cre/*loxP*-mediated gene switching mechanism.

## 3. Discussion

We have reported that liver-specific gene switching can be induced when Cre/*loxP*-related components are introduced via the tail vein [14]. Unfortunately, this event was transient, given that the nucleic acids used in that study were all plasmid DNA. To determine the function of a GOI in vivo, persistence of its expression appears to be a prerequisite. For this, there is a need for chromosomal integration of the GOI when nucleic acids are intravenously delivered. 

*piggy*Bac is one of the transposon-based gene delivery systems [28,29], which has been found to be useful for allowing efficient chromosomal integration of a GOI in cultured cells and for efficient transgenesis in mice [20,23,25,28,30,31]. *piggy*Bac-based gene delivery is very simple: one can put PB transposase expression vector and transposon vectors carrying GOI flanked by the two inverted terminal repeats (ITR) sequences (referred to as “PB acceptor” in this paper). When they are placed inside a cell, transposase binds to the ITR to allow the GOI alone to be integrated into host chromosomal sites that contain the TTAA sequence, which is duplicated on the two flanks of the integrated fragment [17,32]. Unfortunately, little is known about whether this system is effective in vivo. Saridey et al. demonstrated that a single injection of plasmid-based PB transposons via the tail vein confers long-term (approximately 300 days after gene delivery) expression of the GOI (encoding luciferase) in the liver and lung of mice, suggesting the chromosomal integration of the GOI [33]. Similar results were also provided by other groups who used repeated intravenous injections of PB transposons [27] or intravenous injection of hybrid PB/viral vectors [34]. Notably, Nakanishi et al. reported continuous expression of a GOI in the liver over 2 months after HGD [27], which appears to be in consistent with our present results (see Figure 1C). For example, intravenous introduction of plasmid-based PB transposons (pTrans and pT-EGFP) and non-PB plasmid (ptdTomato) resulted in the persistent expression of EGFP-derived fluorescence in the liver during the period of 2 to 56 days after HGD, while the expression of tdTomato-derived fluorescence only continued for up to 7 days (see Figure 1C). This was also confirmed by PCR analysis, showing that the introduced pT-EGFP-derived transgene was still detectable in the liver sampled 56 days after HGD, while ptdTomato-derived DNA was not (see Figure 1D). These findings suggest the chromosomal integration of GOI (pT-EGFP) in murine hepatocytes. 

In our previous study, we demonstrated the liver-specific expression of a GOI in mice after intravenous administration of plasmid DNA such as a plasmid pTR/NCre and a plasmid carrying a *loxP*-flanked gene cassette [14]. In this case, other organs such as kidney and lung did not show any recombination of introduced floxed sequences, and only liver showed Cre-mediated excision [14]. This allowed us to use DT-A-based genetic ablation of hepatocytes using this in vivo tissue-specific gene switching system. DT-A is known to be a potent protein leading to cell death through the inactivation of peptide elongation factor [35]. In our previous experiment, the intravenous injection of an expression vector that confers the expression of DT-A under a systemic promoter caused renal lesions due to the destruction of glomerular epithelial cells [36]. Furthermore, the mating of Tg mouse lines called CETD (carrying *CAG*, loxP-flanked *EGFP-CAT* gene, and *DT-A* gene) with a Tg line (carrying *NCre* gene) caused embryonic lethality in bigenic fetuses, as a result of removal of the *loxP*-flanked sequence and subsequent expression of DT-A [37]. Thus, HGD with PB vectors (pTrans and pT-CETD (in which the CETD component is surrounded by the PB acceptors; see Figure 2A)) should lead to the generation of hepatocytes showing long-term expression of EGFP from chromosomally integrated pT-CETD. In fact, PCR analysis of genomic DNA isolated from the liver of mice 28 days after HGD with pT-CETD and pTrans showed the presence of *EGFP* cDNA (included in the introduced pT-CETD) (see Figure 2C). However, when these pT-CETD-incorporating mice were next subjected to HGD with a solution containing pTR/NCre or pTR/lacZ, and 28 days later their liver was analyzed for the presence of recombined pT-CETD (see upper panel of Figure 2D), mice injected with pTR/NCre (but not pTR/lacZ) had a fragment (corresponding to the *DT-A* gene) showing evidence of Cre-mediated gene switching. This means the pT-CETD-incorporated hepatocytes begin to express DT-A when they are transfected with a NCre expression vector. Since DT-A is a potent protein leading to cell death, as mentioned above, the PCR-amplified band may have been derived from hepatocytes just showing Cre-mediated recombination. In this case, the cell showing recombination appears to be healthy because DT-A is not sufficiently expressed in that cell, probably due to pT-CETD potentially having been integrated into a transcriptionally inactive chromosomal region. 

In this study, we used a total of six mice for the induction of Cre/*loxP*-based DT-A-mediated ablation of hepatocytes (see Figure 2B). They were subjected to HGD with pTrans and pT-CETD approximately 1 month prior to the second gene delivery with TR-directed vectors. The six mice were divided equally into two groups: one was an experimental group (HGD with pTR/NCre) and the other was control-1 group (HGD with pTR/lacZ). The administration of pTR/NCre into the pT-CETD-incorporated mice caused abnormality in serum biochemical parameters in the initial stage of the second gene delivery. For example, they showed increases in the levels of AST and ALT (see Figure 3), suggesting the occurrence of liver injury. A rapid increase in the level of AST is known to be correlated with acute hepatitis [38]. Notably, the level of ALT in relation to that of AST increased with time (Figure 3), suggesting a transition into chronic hepatitis. The amount of albumin, a protein mainly produced in the liver, was reduced with a peak 7 days after the second gene delivery, and the level of total bilirubin gradually increased around 7 days and more after the second gene delivery (Figure 3). Inspection of H&E-stained specimens demonstrated that, although no sign of fibrosis was noted, several pathological abnormalities (such as many irregular patchy areas of necrosis with infiltration of inflammatory cells; see Figure 4a) were seen in the experimental group. In contrast, in the control-1 and -2 groups, no obvious abnormality was noted (see Figure 4b,c). Based on these findings, the mice (having lesions in the liver after the Cre-mediated ablation of hepatocytes) created in this study were judged to have several liver lesions, although they had not developed full hepatitis yet. These mice appear to be useful as models for liver injury to perform basic research on the pathogenesis of the liver and to develop therapeutic methods for liver regeneration. Notably, Zhang et al. reported that a vast majority of the hepatocytes (~80%) can be replaced by human cells in severe combined immunodeficient (SCID) mice that have previously undergone diphtheria toxin (DT)-mediated hepatic ablation [39]. Briefly, in this model, liver injury was induced by intravenous injection of DT into the Tg mice (Alb-TRECK/SCID) expressing the DT receptor in a liver-specific manner. In these mice, the injected DT binds to the DT receptor expressed on the surface of hepatocytes, leading to selective cell death. When immature human hepatocytes were transplanted into Alb-TRECK/SCID mice with liver injury, they could successfully repopulate their livers, thereby creating “humanized mice.” In this context, the combined use of PB and HGD to induce liver injury could be useful to generate “humanized mice” to explore in vivo drug metabolism and drug-drug interactions, as suggested by Katoh and Yokoi [40].

The PB system is known to enable the simultaneous introduction of multiple (over 10) gene constructs [41] with relatively high efficiency. This system principally allows the chromosomal integration of multiple genes (such as those involved in liver regeneration) at once and furthermore allows the regulated expression of a GOI, when used in combination with the Cre/*loxP* system, as shown here. PB enables the seamless removal of the PB transposons through introduction of a PB transposase expression vector into the transposon-incorporating cells [25]. If this can be performed efficiently, the system presented here would be useful for basic study towards in vivo genetic manipulation and analysis of hepatocyte function without the need to produce Tg animals. 

A major potential limitation of this present system is that EGFP-derived fluorescence in the liver achieved after HGD using pTrans and pT-EGFP could not be maintained for a long time because, at the early stage post-transfection, fluorescence was distinctly observed throughout the liver, but it was later greatly attenuated, as shown in Figure 1C. This means that almost all of the PB vectors successfully delivered into the liver cannot be efficiently integrated into the host hepatocyte’s chromosomes, although a few PB transposons had been thought to be integrated into host cells. This may be because PB system requires 2 independent DNA elements (PB transposase expression vector and transposon vectors carrying GOI flanked by PB acceptor) into a single cell, or in the transposon-based gene delivery system, the overexpression of transposase often hampers efficient gene expression of a GOI, which is now called “overproduction inhibition,” the severity of which depends on the cell type used for transfection [42]. This may be solely due to the nature of PB transposase itself: it mediates the chromosomal integration of transposons, but also mediates the excision of chromosomally integrated transposons, as mentioned above. In this context, it is necessary to determine adequate concentrations of a transposase expression vector to be used for gene delivery into the liver.

One of the characteristics of our method is that it permits a timely controlled liver-specific gene switching of a GOI in vivo using the Cre/*loxP* system. Cre/*loxP*-based in vivo gene switching systems using fluorescent reporters have already been reported [43,44,45]. However, these systems suffer from the limitation of relying on the use of genetically modified mice. Currently, it is commonly believed that the most reproducible way to achieve long-term expression of a GOI (whose expression is induced after gene switching) in the specific tissue of a non-Tg mouse is through chromosomal integration. To obtain this, we employed the PB system, which allows chromosomal integration of an exogenous DNA of approximately 10 kb in size [20]. It has been reported that the expression of a chromosomally integrated GOI via the PB system continued over 300 days [33], and that HGD-mediated gene delivery of a naked plasmid in the liver confers the GOI an expression lasting for approximately 4 weeks [2,46].

Lastly, in this study we achieved chromosomal integration and long-term expression (approximately 2 months) of an exogenous GOI in the liver after HGD-mediated gene delivery (Figure 1C,D). Sustained expression of GOIs in cells transfected with plasmid DNA was previously obtained using a number of methods employing (1) CpG-less plasmid vectors [47,48]; (2) episomal vectors [49,50]; (3) hybrid promoters, in which the CMV enhancer is combined with promoters of the human mucin I (*MUC-I*) gene [51], ubiquitin gene [52], or human elongation factor 1α (*EF1α*) gene [53]; (4) liver-specific vectors (pLIVE) consisting of multiple cloning sites between two introns [54,55,56]; (5) minicircle DNA [57,58]; and (6) phiC31 integrase vector [59,60]. Additional experiments will thus be aimed to investigate whether these methods can be applied to further prolong gene expression in HGD-modified cells.

## 4. Materials and Methods

### 4.1. Plasmid Vectors

pTrans (pCX-mPB; Figure 1A) is a plasmid vector allowing expression of the PB transposase under control of the chicken β-actin promoter-based *CAG* [61]. Two PB expression plasmids pT-EGFP (Figure 1A) and pT-CETD (Figure 2A) were generated using pPB (pPB-MCS-P5), a plasmid carrying two PB acceptors with inverted repeats, as a basal plasmid. pT-EGFP carries an *EGFP* gene expression unit (CAG + *EGFP* cDNA + poly(A) sites) [62]. pT-CETD confers gene switching from *EGFP* cDNA to the *DT-A* gene when Cre expression occurs, as previously discussed [37]. DT-A is known to kill cells by ribosylating translation elongation factor 2 (EEF2) and inhibiting protein synthesis [35]. It is estimated that a single molecule of the natural protein is sufficient to kill a cell [63]. DT-A has been used to selectively ablate tissues and cells in Tg animals [64,65,66,67,68]. The plasmid pTR/NCre (Figure 2A) enables liver-specific expression of NCre (Cre gene associated with a sequence for a nuclear localization signal at its 5′ end) under the transcriptional control of a liver-specific mouse transthyretin (i.e., prealbumin) promoter [14]. As control plasmids, ptdTomato (Figure 1A), which confers systemic expression of tdTomato under CAG, and pTR/LacZ (Figure 2A), which confers liver-specific expression of lacZ, were used.

### 4.2. Mice

Institute of Cancer Research (ICR) male mice (five-week-old; Clea Japan, Inc., Tokyo, Japan) were used. They were kept under a 12 h light/12 h dark schedule (lights on from 0700 h to 1900 h) and allowed food and water ad libitum. In the experiment, mice were used under sufficient anesthesia after the intraperitoneal (IP) injection of the combination of three anesthetics (medetomidine (0.75 mg/kg; Nippon Zenyaku Kogyo Co. Ltd., Fukushima, Japan), midazolam (4 mg/kg; Sandoz K.K., Tokyo, Japan) and butorphanol (5 mg/kg; Meiji Seika Pharma Co., Ltd., Tokyo, Japan)). Recovery of the mice from anesthesia was performed by the IP injection of atipamezole (3.75 mg/kg; Nippon Zenyaku Kogyo Co., Ltd., Fukushima, Japan), an antagonist of medetomidine, followed by warming with an electric plate warmer.

All animal experiments were performed at the National Defense Medical College (Saitama, Japan), in accordance with the guidelines of *National Defense Medical College Committee on Recombinant DNA Security*, and approved by *The Care and Use of Laboratory Animals* (permission no. 12002, valid from 3 July 2012 to 31 March 2015; and no. 15002, valid from 13 July 2015 to 31 March 2018). All efforts were made to minimize the number of animals used and their suffering.

### 4.3. In Vivo Gene Delivery by the Intravenous Injection of Plasmids

For in vivo gene delivery via the tail vein, we employed HGD as previously reported [14,69]. In brief, mice were injected with a plasmid DNA-containing TransIT-EE Hydrodynamic Delivery Solution (Takara Bio Inc., Shiga, Japan; hereafter referred to as TransIT-EE) (one-tenth of the weight/volume (in mL) per mouse; for example, 3 mL/30 g of a mouse) by a syringe (3 mL Luer lock type; Nipro, Inc., Osaka, Japan) fitted with a 27-gauge needle (Nipro, Inc.). Injections were performed at a constant injection speed via the tail vein and completed within 10 s. The injection was performed by the same researcher in order to avoid artifactual effects in each experiment. In the experiment for long-term gene expression in murine liver (Figure 1B), we injected 3 mL of TransIT-EE containing pT-EGFP (10 μg), pTrans (5 μg), and ptdTomato (10 μg) per mouse (30 g). In the experiment for the creation of disease models for liver dysfunction (Figure 2B), we injected 3 mL of TransIT-EE containing pT-CETD (10 μg) and pTrans (5 μg) per mouse (30 g). One month later, the mice were subjected to a second HGD with 3 mL of TransIT-EE containing 10 μg of pTR/NCre (as an experimental group; a total of three mice were used) or 10 μg of pTR/LacZ (as control-1 group; a total of three mice were used). Males were subjected to mock injection with 3 mL of TransIT-EE alone or no-injection (as control-2 group (Mock/Intact); a total of three mice were used respectively).

### 4.4. Biochemical Examination

Liver function was evaluated using representative biochemical parameters for hepatic disorder. On 0, 2, 7, 14, and 28 days after the second gene delivery to the pT-CETD/pTrans-treated mice with pTR/NCre (experimental group) or pTR/lacZ (control-1 group), the mice were subjected to blood collection (50 μL) to obtain serum. Similarly, blood from mice injected with TransIT-EE alone (control-2 group) (shown in Figure 2B) was collected. The collected serum was then subjected to measurement of the levels of AST, ALT, albumin, and total bilirubin using FUJI DRI-CHEM slide system (DRI-CHEM 4000V; FUJIFILM Co., Tokyo, Japan), in accordance with the manufacturer’s instructions. 

### 4.5. Microscopic Observation

In the experiment for long-term gene expression in murine liver (as shown in Figure 1B), whole livers of treated mice were dissected 2, 7, 28, and 56 days after HGD, and immediately transferred onto ice. To detect EGFP/tdTomato-derived fluorescence on a liver’s internal area of the right median lobe, it was cut in half using a microtome blade (Feather Safety Razor Co., Ltd., Osaka, Japan) and then examined under a fluorescence microscope (BZ-8000; Keyence Co., Osaka, Japan). In the experiment for the creation of mice with liver dysfunction (as shown in Figure 2B), whole liver was dissected 28 days after the second gene delivery in the pT-CETD/pTrans-treated mice with pTR/NCre (experimental group) or pTR/lacZ (control-1 group) and immediately transferred onto ice. Similarly, liver was dissected from mice 28 days after administration with TransIT-EE alone (control-2 group). For histological analysis, some of the samples were then fixed with 10% formaldehyde in PBS and subjected to standard histological processing by using H&E staining. The specimens were inspected using the light microscopy mode of a BZ-8000 fluorescence microscope. 

### 4.6. Detection of Transgenes by PCR

Genomic DNA from the transfected liver was isolated as previously described [14]. Genomic PCR was performed, as previously described [37], in a reaction mixture with a total volume of 10 μL with each of the following primer sets (Table 1): (1) EGFP-10S/EGFP-10RV producing 384-bp fragments from the *EGFP* cDNA in pT-EGFP and pT-CETD [41]; (2) TDR-3S/TDR-3RV producing 206-bp fragments from the *tdTomato* cDNA in ptdTomato [41]; (3) β-gl-1S/DTA-2RV yielding 413-bp products corresponding to the recombined form of pT-CETD (a product generated after the Cre-mediated excision of pT-CETD) [37]; and (4) mEx4-S/mEx4-RV producing 390-bp fragments from endogenous mouse α-1,3-galactosyltransferase (α-GalT) [70], for confirmation that the samples loaded into the gel were derived from PCR using the same amounts of DNA. Five nanograms of plasmid DNA (pT-EGFP, pT-CETD, and ptdTomato) was concomitantly subjected to PCR as positive controls. Four microliters of each of the resulting PCR products was separated on a 4% agarose gel and then stained with ethidium bromide (EtBr) for DNA visualization.

### 4.7. Statistical Analysis 

For evaluation of the data of the biochemical examinations and weights of the mice in each condition, the data are presented as mean ± standard deviation (SD). Statistical analysis was performed using the unpaired *t*-test and one-way factorial analysis of variance (ANOVA). Scheffe’s post hoc test was used for multiple comparisons. *P* values were calculated using the GraphPad PRISM 5 for Windows software (GraphPad Software, Inc., La Jolla, CA, USA). A *P* value of less than 0.05 was considered to indicate statistical significance.

## 5. Conclusions

Using PB and HGD, we successfully achieved the long-term expression of a GOI in murine liver and Cre/*loxP*-based gene switching which is liver-specific and can be performed at any time the researcher wants. The expression of GOI in murine liver was still discernible even approximately two months after HGD. When we applied these technologies to produce liver disease model mice, the resultant mice exhibited altered biochemical parameters and pathological abnormality, although they did not suffer from full hepatitis yet. This present gene-based technology should also be useful for establishing an in situ manipulation system to assess liver function without the need to produce Tg animals.

## Figures and Tables

**Table 1:** Nucleotide sequences of primers used in this study.

Name of Primer	Sequence (5′–3′)
EGFP-10S [41]	CCT GAA GTT CAT CTG CAC CAC
EGFP-10RV [41]	GTT GTG GCG GAT CTT GAA GTT
TDR-3S [41]	CCC GTA ATG CAG AAG AAG ACC
TDR-3RV [41]	GTG ATG TCC AGC TTG GTG TCC
β-gl-1S [37]	TGT GCT GTC TCA TCA TTT TGG
DTA-2RV [37]	GCG AGA ACC TTC GTC AGT CCT
mEx4-S [70]	GCA AAT GTG GAT GCT GGG AAC
mEx4-RV [70]	ACA GTT TTA ATG GCC ATC TGG

## Supplementary Materials

Supplementary materials can be found at http://www.mdpi.com/1422-0067/19/11/3452/s1.

## Abbreviations

α-GalT	α-1,3-galactosyltransferase
ALT	alanine transaminase
ANOVA	one-way factorial analysis of variance
AST	aspartate transaminase
CAG	unit comprising cytomegalovirus enhancer, chicken β-actin promoter, second intron of rabbit *β-globin* gene
CAT	chloramphenicol acetyltransferase
CETD	unit comprising CAG, loxP-flanked EGFP cDNA, CAT gene, *DT-A* gene, and poly(A) sites
CMV	cytomegalovirus
DT	diphtheria toxin
DT-A	cDNA for diphtheria toxin-A chain
EEF2	ribosylating translation elongation factor 2
EGFP	enhanced green fluorescent protein
EtBr	ethidium bromide
GOI	genes of interest
GOT	glutamic oxaloacetic transaminase
GPT	glutamic pyruvic transaminase
H&E	hematoxylin & eosin
HGD	hydrodynamics-based gene delivery
ICR	Institute of Cancer Research
IP injection	intraperitoneal injection
iPS cell	inducible pluripotent stem cell
ITR	inverted terminal repeat sequence
lacZ	β-galactosidase
NC	negative control
NCre	unit comprising nuclear localization signal gene, *Cre* gene
pA,	poly(A) sites
PB	*piggy*Bac
PB acceptor	acceptor site in PB system
PC	positive control
PCR	polymerase chain reaction
pT	PB-based plasmid
SCID	severe combined immunodeficient
SD	standard deviation
tdTomato	tandem dimeric Tomato
TR	transthyretin promoter
TSTA	two-step amplification
