# Supplementary figures and images for: Intravenous Delivery of piggyBac Transposons as a Useful Tool for Liver-Specific Gene-Switching
Source: Int J Mol Sci. 2018 Nov 2;19(11):3452. doi: 10.3390/ijms19113452 (PMC6274756; doi:10.3390/ijms19113452)

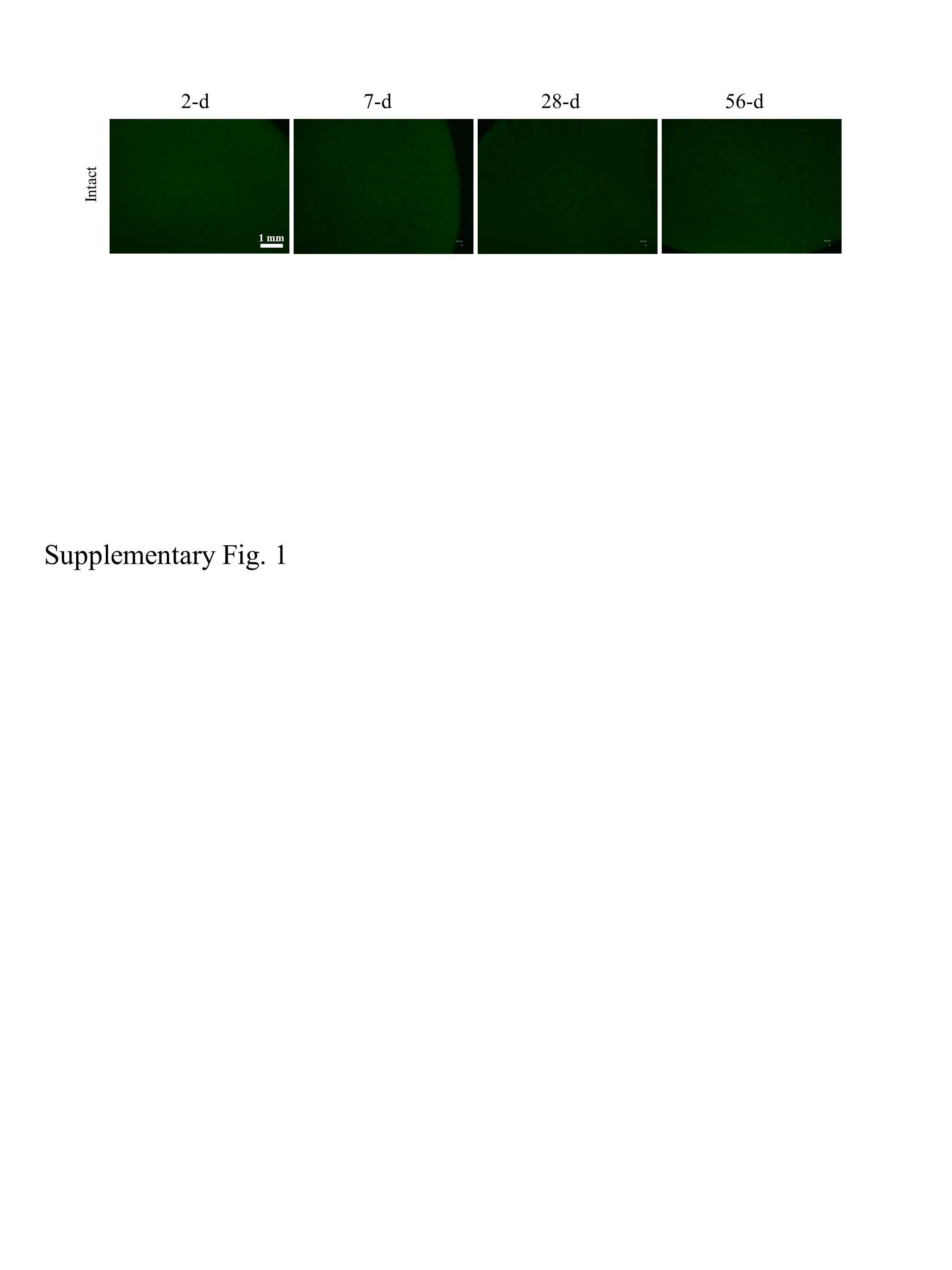

Supplement: Supplementary file 1 [file ijms-19-03452-s001.jpg]
